# Therapeutic Perspectives of Adenosine Deaminase Inhibition in Cardiovascular Diseases

**DOI:** 10.3390/molecules25204652

**Published:** 2020-10-12

**Authors:** Barbara Kutryb-Zajac, Paulina Mierzejewska, Ewa M. Slominska, Ryszard T. Smolenski

**Affiliations:** Department of Biochemistry, Medical University of Gdansk, Debinki 1 St., 80-211 Gdansk, Poland; paulina.mierzejewska@gumed.edu.pl (P.M.); ewa.slominska@gumed.edu.pl (E.M.S.)

**Keywords:** adenosine deaminase, ADA, inhibition, therapy, inflammation, atherosclerosis, myocardial infarction, thrombosis, hypertension, type II diabetes mellitus

## Abstract

Adenosine deaminase (ADA) is an enzyme of purine metabolism that irreversibly converts adenosine to inosine or 2′deoxyadenosine to 2′deoxyinosine. ADA is active both inside the cell and on the cell surface where it was found to interact with membrane proteins, such as CD26 and adenosine receptors, forming ecto-ADA (eADA). In addition to adenosine uptake, the activity of eADA is an essential mechanism that terminates adenosine signaling. This is particularly important in cardiovascular system, where adenosine protects against endothelial dysfunction, vascular inflammation, or thrombosis. Besides enzymatic function, ADA protein mediates cell-to-cell interactions involved in lymphocyte co-stimulation or endothelial activation. Furthermore, alteration in ADA activity was demonstrated in many cardiovascular pathologies such as atherosclerosis, myocardial ischemia-reperfusion injury, hypertension, thrombosis, or diabetes. Modulation of ADA activity could be an important therapeutic target. This work provides a systematic review of ADA activity and anchoring inhibitors as well as summarizes the perspectives of their therapeutic use in cardiovascular pathologies associated with increased activity of ADA.

## 1. Introduction

Adenosine deaminase (ADA, EC 3.5.4.4), known as adenosine aminohydrolase, is a key enzyme engaged in purine metabolism that irreversibly converts adenosine to inosine or 2′deoxyadenosine to 2′deoxyinosine [1]. The amino acid sequence of ADA is highly conserved between species and it has a wide phylogenetic distribution from bacteria to humans [2,3]. In human tissues, adenosine deaminase is present as two isoenzymes: ADA1 and ADA2. [4] ADA1 constitutes the majority of ADA activity and it is present in virtually all tissues, with the highest levels in lymphoid cells, red blood cells and endothelium [4,5]. In addition to cytosolic form, ADA1 can be found as an ecto-enzyme binding to the cell surface by CD26 protein or adenosine receptors [6]. The second iso-enzyme (ADA2) belongs to the adenosine deaminase growth factor family and it is found with ADA1 mainly in monocytes/macrophages [7]. Zavialov et al. revealed that similarly to ADA1, ADA2 can be bind to the cell surface by proteoglycans or adenosine receptors [8]. As both isoenzymes of ADA maintain their catalytic activities after cleavage from the plasma membrane of intact cells submitted to stressful conditions, this makes them capable of deaminating adenosine into inosine in the soluble fraction of a given tissue, even if it is located away from the originating cell. [9] It has been shown that both isoenzymes, but with a predominance of ADA2, are present in human plasma in the soluble forms [10]. Plasma of rat and mouse are totally devoid of ADA2, where entire adenosine deaminating activity derives from ADA1 [7]. The differentiation of ADA1 and ADA2 isoenzymes has been possible since ADA1 activity is inhibited by (+)-erythro-9-(2-hydroxy-3-nonyl)adenine (EHNA) and its derivatives, whereas ADA2 activity is not affected by these inhibitors [11]. The inhibition of ADA1 by EHNA-derived compounds provides evidence of the difference in binding sites of EHNA in two ADA isoenzymes: the hydrophobic site on ADA1 that is required for complex formation with the aliphatic chain of EHNA is probably absent in ADA2 [12]. In humans, the ADA1-like domain of the ADA2 iso-enzyme shares about 70% of its amino acid similarity with the ADA1 protein [13].

Intracellularly, adenosine deaminase plays a significant and diverse metabolic role. The importance of normal ADA activity levels is demonstrated by the effect of a genetic deficiency of this enzyme, which is associated with a form of severe combined immunodeficiency disease (SCID). [14] Due to the lack of ADA activity, 2′deoxyadenosine accumulates and is converted to 2′-deoxy-adenosine-5′-triphosphate (dATP), which inhibits ribonucleotide reductase (RNR), a crucial enzyme in DNA synthesis that follows disrupted T-cell development [15]. In physiology, concentration of adenosine, the second substrate for ADA, is maintained inside the cell on the low level by adenosine kinase with Km value ~ 1 µM [16]. However, during inflammation or hypoxia, adenosine is produced from intracellular ATP under conditions of low energy charge. AMP, which originates from ATP degradation is rapidly transformed to adenosine that is not immediately deaminated to inosine due to high Km of ADA (25–100 µM) [17]. This results in temporary accumulation of adenosine, which is vigorously exported out the cell via equilibrative nucleoside transporters (ENTs) [18].

**Figure 1:**
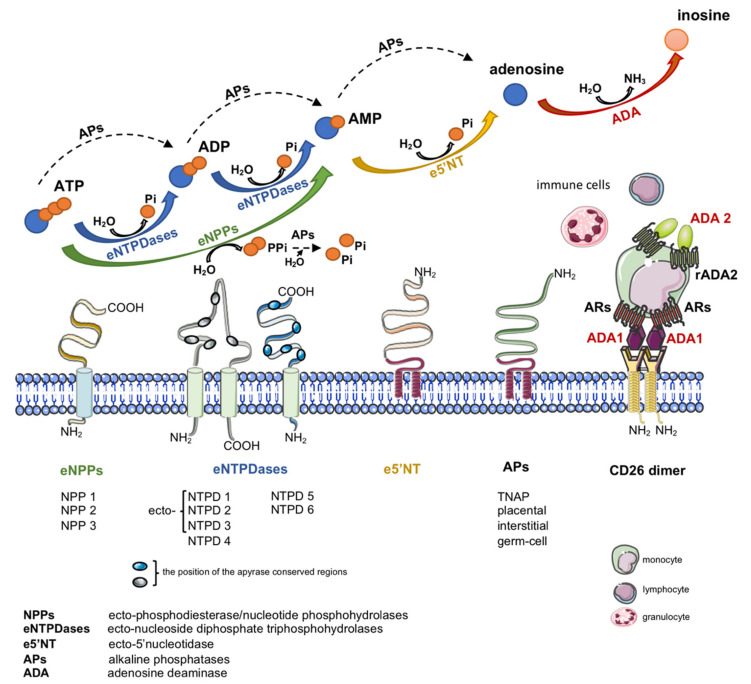
Enzymes engaged in extracellular metabolism of adenine nucleotides and adenosine. Dual role of adenosine deaminase as the adenosine-degrading enzyme and co-stimulatory molecule.

Another important source of extracellular adenosine is the hydrolysis of adenine nucleotides by cell-surface ecto-nucleotidases (Figure 1) [19]. Nucleotides are released to the extracellular compartment in response to cellular stress via lytic and non-lytic mechanisms [20]. Therefore, together with nucleotide-degrading ecto-enzymes and transmembrane adenosine transport, eADA plays an essential role in the regulation of extracellular adenosine concentration.

Adenosine is a ubiquitous molecule with key regulatory and cytoprotective mechanisms. Among a plethora of physiological actions, extracellular adenosine has an important role in cardiovascular system homeostasis [21]. It engages members of the G-protein coupled adenosine receptor (AR) family to mediate mostly beneficial adaptive and acute responses within all constituent cells of the heart and vasculature. In this way the four AR subtypes-A_1_, A_2A_, A_2B_, and A_3_Rs-regulate myocardial contraction, heart rate and conduction, adrenergic control, coronary vascular tone, cardiac and vascular growth, inflammatory-vascular cell interactions, and cellular stress-resistance, injury and death [22]. All subtypes of AR are characterized by seven transmembrane domains connected by three intracellular and extracellular domains. At the extracellular level, the N-terminus presents specific glycosylation sites, while at the intracellular side, the C-terminus contains phosphorylation and palmitoylation sites, important for receptor desensitization [23]. Each AR has unique cell and tissue distribution, secondary signaling transductors, affinity for adenosine, and physiological effects. In cardiovascular system, all AR subtypes were demonstrated in endothelium, vascular smooth muscle cells, cardiomyocytes, valve cells and many others [4,22,24]. A_1_R and A_3_R predominantly activate heterotrimeric G proteins belonging to the Gα i/o family, which inhibit cAMP production by adenylate cyclase, in contrast A_2A_R and A_2B_R predominantly activate Gαs family members, which stimulate cAMP production [25]. The A_1_, A_2A_ and A_3_ receptors possess high affinity for adenosine while the A2B shows relatively lower affinity for this nucleoside. During normal conditions, the extracellular adenosine accumulation is limited to 30–300 nM range activating only high-affinity AR [26]. Mounting evidence indicates that extracellular adenosine levels increase dramatically to micromolar concentrations that activate low-affinity receptors in tissues submitted to stressful conditions, such as ischemia, hypoxia, and inflammation, being a key protective and anti-inflammatory mechanism [27]. In addition to adenosine, it has been shown that other molecules can non-selectively activate adenosine receptors with micromolar affinities, including natural plant alkaloids such as caffeine, theophylline and their derivatives [28]. Particularly interesting is the fact that also inosine can interact with AR, which emphasizes the importance of eADA as controlling the ratio between extracellular adenosine and inosine [29]. From one side, inosine at micromolar concentrations expressed immunomodulatory and anti-inflammatory effects via A_1_R, A_2A_R and A_3_R [30,31]. While from the other, the opposed effects of inosine and adenosine have been demonstrated. In the study of Herman-de-Sousa et al. [32] adenosine favored normal collagen production by human subcutaneous fibroblasts via A_2A_R, whereas, inosine via A_3_R stimulated inappropriate dermal remodeling, providing ADA inhibition as a therapeutic target to prevent connective tissue disorganization. It is worth noting that inosine at micromolar concentrations stimulates the receptors with high affinity for adenosine, which may be a compensatory anti-inflammatory mechanism in the case of increased eADA activity. In addition, increased eADA expression and hence the amplified association of eADA with AR can allosterically modulate agonist affinity and efficacy [33].

Except its enzymatic function, cell-surface eADA plays a significant extra-enzymatic role in the interactions between cells that expressed ADA-anchoring proteins on their surfaces (Figure 1). Indeed, there is an evidence of the formation of trimeric complexes dipeptidyl peptidase IV (CD26)-ADA-A_2A_R involving two cells [6]. This co-stimulatory and cell-to-cell connecting actions of eADA along with its activity regulate many cellular processes related to proliferation and differentiation, which affect pathological conditions associated with cardiovascular diseases such as endothelial activation and dysfunction, inflammation, myocardial ischemia-reperfusion injury or coagulation disorders. Since these pathologies are associated with ADA overexpression, the inhibition of its activity as well as binding to the surface proteins exhibit an attractive therapeutic potential in cardiovascular diseases. There are several groups of ADA inhibitors and some of them are presently used in clinical practice, however they still show side effects. Therefore, new ADA inhibitors that are devoid of cytotoxic effects are currently intensively studied. Based on above considerations, the present article has been conceived to provide a systematic review of compounds inhibiting ADA activity and its interactions with cell surface proteins as well as to summarize current information on the cardiovascular pathologies with ADA overactivity. Particular attention has been paid to the involvement of ADA in the pathophysiology of relevant cardiovascular diseases and therapeutic potential of its pharmacological inhibition.

## 2. Structure of Adenosine Deaminase (ADA)

Three-dimensional structures as well as molecular catalysis mechanism of the complex adenosine deaminase/inhibitor have been demonstrated by computational [34], crystallographic [35] and mutagenic analysis [36].

ADA is considered as triosephosphate isomerase (TIM)-barrel or (β/α)_8_-barrel structure, which consists of eight peripheral α-helices that surround the central eight parallel β-strands. The TIM-barrel structural scaffold occurs ubiquitously in nature and is seen in many different enzyme families. Many TIM-barrel proteins, including ADA, contain metal ions at active sites [37]. In fact, ADA has a Zn^2+^ ion in its active site. Zn^2+^ ion has key role in its biological function and regulation but also is necessary for the protein stabilization [38]. The product of ADA gene consists of 363 amino acids. Mammalian ADA is evolutionarily conserved from bacteria to humans. Human and bovine ADA share approximately 88% amino acid sequence identity [39]. However, despite the high degree of amino acid sequence conservation among various species, the human enzyme has a longer carboxy terminal region than the murine and bacterial homologues [40]. Understanding the structure of the enzyme has provided detailed information of the amino acids in the active site responsible for the catalytic mechanism. Briefly, an initial addition of the stereospecific hydroxyl group to the C6 position of the adenosine to create the tetrahedral transition-state intermediate and the final elimination of ammonia to form inosine. Zn^2+^ is the key player acting as strong electrophile and activating the water attacks to the substrate [41]. A glutamic acid (Glu) directly interacts with the heterocyclic nitrogen atom (N1) of adenine ring of the adenosine and is involved in donation of a proton to N1, reducing thereby the double bond character between N1 and C6 and making the C6 more susceptible to the hydroxyde nucleophilic attacks. Moreover, a histidine residue is also involved in the formation but also stabilization of the attacking hydroxide derived from the initially Zn^2+^-bound water molecule. Furthermore, the carboxylate group of an aspartic acid (Asp) assists in properly orienting the hydroxide oxygen in line to the C6 addition. The final step consists in ammonia elimination [35]. Tryptophan residues are not directly involved in the catalytic mechanism of ADA. However, the intrinsic fluorescence was disturbed after binding with inhibitors. After ultraviolet irradiation and chemical modification of ADA derived from calf intestine, the transformation of fluorescent tryptophan residues is observed together with the reduced enzymatic catalytical activity and confirms the probable location of tryptophans near the binding site of the enzyme [42]. ADA inhibitors and substrates, EHNA and adenosine analogs, were capable to protect tryptophan residues against chemical modification—*N*-bromosuccinimide oxidation—and this effect was correlated with the enzyme activity protection [43]. However, the role of the tryptophan residues in the catalytic mechanism of ADA requires further studies.

Two isoenzymes, but three molecular isoforms of human ADA are known: two ADA1 forms—a low molecular weight form (34–43 kDa) and high molecular complex with the ADA-binding protein (280–300 kDa), and ADA2 (110 kDa) [44]. ADA1 and ADA2 differ in kinetic and immunochemical properties. They appears to be encoded by a separate genetic locus, ADA1 by gene named ADA1 and ADA2 by CECR1 [45]. The Km of ADA2 for Ado is 1.48 mM and is approximately 15 times higher than Km of ADA1 (< 0.1 mM). Km values for Ado and 2′-dAdo are almost the same [46]. The study of Andreasyan et al. proved that both ADA1 and ADA2 are characterized by similar involvement of Zn^2+^ ions in the active center. Moreover, the pH-profile and pKa values of ADA2 on the acidic side, 4.1 and 4.5, and on the basic side, 7.8, were evaluated. The observed acidic side pKa values suggest that Asp with pKa 3.9 and Glu with 4.3 are participating in adenosine deamination catalyzed by ADA2 [12]. The same was confirmed for ADA1 by crystallographic analysis [47]. Furthermore, the lower pKa value for ADA2 in comparison with the value for ADA1 (5.6) provides the evidence for the more substantial role of above mentioned amino acids in ADA2 catalyzed reaction than in the case of ADA1 [39]. 

## 3. Inhibitors of ADA Activity

Typically, ADA inhibitors are classified into transition-state inhibitors, ground-state compounds and non-nucleoside inhibitors as shown in Table 1 [48]. The structure of transition-state inhibitors mimics that of the tetrahedral intermediate, which forms during the deamination process catalyzed by ADA. Ground-state compounds are similar to the endogenous substrate, adenosine. The third class of derivatives, non-nucleoside inhibitors, have been recently added to previous ones, and specifically include a series of imidazole-4-carboxamides, design and developed by Terasaka and co-workers at Fujisawa Pharmaceutical Company [49]. Moreover, many other compounds including drugs or plant phenolics, such as flavonoids potently inhibit ADA activity. Above classes of ADA inhibitors will be presented in the following sections of this review as well as the molecules that interfere with the interaction between ADA and cell-surface proteins will be disclosed.

### 3.1. Transition-State Inhibitors

#### Coformycin and Deoxycoformycin Analogs

About 40 years ago, coformycin (CF) was isolated from a culture of Gram-positive aerobic bacteria, *Nocardia interforma* and *Streptomyces kaniharaensis*, while 10 years later, the 2-deoxy derivative of coformycin (2′deoxycoformycin, dCF, pentostatin), was extracted from a *Streptomyces antibioticus* fermentation [50,51]. These two compounds represent the main examples of transition-state inhibitors that potently inhibit ADA activity with Ki values of 10 and 2.5 pM, respectively. Their efficacy has been attributed to extremely tight-binding, long and almost irreversible interaction with the enzyme [80]. Both these derivatives display a tetrahedral carbon (C8) bearing a hydroxyl group. The stereochemistry at this position significantly affects the potency, being the 8*R*-diastereomer about 107 times stronger than the 8*S* counterpart.

As stated above, ADA activity supports the proliferation of intensely dividing cells by removing 2′deoxyadenosine that cannot be converted to dATP in excessive amounts that disrupts DNA synthesis [15]. Therefore, ADA inhibition provides an efficient immunosuppressive tool and high CF and dCF doses have been proposed for treatment of B- and T-cell malignancies, characterized by increased ADA activity. Since 1991, dCF is successfully used for the treatment of hairy cell leukemia [1]. Additionally, dCF alongside with 2′chloro-2′-deoxyadenosine (cladribine), an adenosine analog that also possesses ADA inhibitory properties [53] and similarly to 2′-deoxyadenosine could be incorporated into mitochondrial and nuclear DNA triggering apoptosis, have been used for the treatment of chronic myelogenous leukemia, cutaneous T-cell lymphoma and chronic lymphocytic leukemia [1], though high doses of dCF are characterized by a relatively high toxicity, especially for central nervous system, kidney and liver, which results from tight-binding interaction with ADA. Moreover, dCF has a well described acid-lability that results in the lack of oral bioavailability and imposes its intravenous administration [81]. The modifications into the structures of CF and dCF have been provided to ensure less toxicity by more reversible ADA inhibitors. However, these manipulations also influenced their inhibitory activity. 4*R*-(1-Hydroxyethyl)-5-methyl-1-β-d-ribofuranosylimidazole that is the effect of CF aglicone structure simplification inhibited ADA activity with Ki value of 61 µM [52]. The substitution of dCF deoxyribose moiety with acyclic sugar as well as the homologation at the C5 position with alkyl chains reduced the tight-binding on ADA about 10^5^ times. 2′-chloro-2′deoxycoformycin, named as adechlorin, isolated from the fermentation broth of *Actinomyces* strain OMR-37 that has the same aglycone of dCF coupled with 2′-chloro-2′deoxyribose was more reversible than dCF and revealed weaker inhibition of ADA with Ki value of 0.53 nM [55]. Another derivative isolated from *Actinomyces* strain OMR-3223, adecypenol includes a carbocyclic sugar and the same aglicone of dCF resulting on a semi-tight binding inhibitor with a Ki 4.7 nM [56].

### 3.2. Ground-State Compounds

#### 3.2.1. Deaza- and Dideazaadenosine Derivatives

Among deaza and dideaza derivatives of adenosine, only 1-deazaadenosine and its 2′deoxy- derivative represent the most potent inhibitors of ADA with Ki values of 0.66 µM and 0.19 µM, respectively [57,58]. It has been investigated that 3-deaza- and 1,3-dideazaadenosine are rather poor inhibitors, while 7-deaza- (tubercidin) and 1,7-dideazaadenosine are fully inactive [82]. 1-Deazaadenosine maintains all features for molecular recognition as a substrate for ADA but due to a lack of N1-protonation that is required for catalytic activity it is not deaminated [83]. The presence of chlorine atom in position 2 resulted in a decreased ADA inhibitory activity. Whereas, introduction of a chlorine atom in this position of substrates produced the compounds more resistant to ADA [84]. Substitution in the N6 position of 2′-deoxyribose derivatives with hydroxyl, methyl and cyclopropyl groups resulted in good inhibitory effects with Ki values 0.25, 1.2 and 5.9 µM, respectively [59]. 3′-Deoxy-1-deazaadenosine and 2′3′-dideoxy-1-deazaadenosine also presented good inhibitory activities for ADA and there were as follows, Ki = 2.6 µM and Ki = 2.2 µM [60]. Interestingly, even though 3-deazaadenosine did not show a significant inhibitory properties for ADA, it has been described as a potent inhibitor and substrate for intracellular enzyme, *S*-adenosylhomocysteine hydrolase (SAHH) [85]. The inhibition of SAHH activity by 3-deazaadenosine may impact on DNA and histones methylation, and thus on epigenetics that might also be involved in the pathophysiology of cardiovascular diseases [86]. Moreover, binding of 3-deazaadenosine to SAHH resulted in the accumulation of *S*-adenosylhomocysteine and *S*-adenosylmethionine as well as the massive production of 3-deazaadenosylhomocysteine in cultured endothelial cells inhibiting their adhesiveness to leukocytes by the effect on decreased ICAM-1 synthesis [87]. Therefore, deaza derivatives of adenosine that do not inhibit ADA also deserve an attention in the preventing of cardiovascular diseases.

#### 3.2.2. EHNA-Like Compounds

Erythro-9-(2-hydroxy-3-nonyl)adenine (EHNA) is formed by adenine coupled to a chiral hydroxynonyl chain in the N9 position. It is a semi-tight ADA inhibitor that at first step, represents a classical competitive inhibition, while later on a sequential rearrangement of the enzyme and inhibitor take place, yielding a tight ADA-inhibitor complex. [88] The *erythro* diastereomer is more active than the *threo* one and Ki value of EHNA ranges 1.6–7.0 nM, depending on the experimental conditions [61].

The modifications of lipophilic hydroxynonyl chain have been optimized and only a few are well tolerated. The chain could be elongated up to C9 and the introduction of a chlorine atom or lipophilic group, like phthalimido resulted in Ki values of 2.7 nM (9′-chloro-EHNA) and 0.95 nM (9′-phthalimido-EHNA) [62]. In turn, hydroxylation at the terminal carbon decreased the affinity confirming the hydrophobic nature of the binding pocket for the aliphatic chain. The addition of a phenyl ring at various position of the side chain exhibited maintained activity only when the phenyl ring has been bridged to the adenine system by at least two carbon atoms [89]. The useful modification of EHNA structure for studying the catalytic mechanism of ADA has been provided by fluorescent derivatives, including epsilon-EHNA with Ki value of 2.8 µM [63].

In order to examine the structural parameters in the purine moiety of EHNA that are critical for inhibitory activity, the deaza analogues of EHNA and the corresponding deaminated derivatives were synthesized and tested. It has been demonstrated that the isosteric monosubstitution of the pyrimidine nitrogen by carbons can be tolerated at the inhibitory binding site. 3-DeazaEHNA and l-deazaEHNA presented good inhibitory activities with with Ki values of 0.01 µM and 0.16 µM, respectively [57,64]. In turn, deaminated nucleoside derivatives, exhibited lower affinity than the corresponding EHNA analogues. This effect has been the most pronounced in EHNA and 3-deazaEHNA (Ki = 0.84 µM vs. Ki = 0.007 µM; Ki = 0.12 µM vs. Ki = 0.01 µM) [57].

The simplification of the chemical structure of EHNA provided a group of erythro-1-(2-hydroxy-3-nonyl)imidazole derivatives. The opening of the pyridine or pyrimidine ring of EHNA led to compounds that maintained good inhibitory activities for ADA. The most potent in this series has been the compound with amido group at C4 (erythro-9-(2-hydroxy-3-nonyl)imidazole-4-carboxamide) with Ki = 0.035 µM, while erythro-1-(2-hydroxy-3-nonyl)imidazole itself exhibited Ki value of 0.90 µM [57,65]. In order to introduce additional simplifications on the structure of these inhibitors, a series of *erythro* and *threo*-9-(2-)hydroxy-3-nonyl azoles have been synthesized and erythro-9-(2-hydroxy-3-nonyl)1,2,4-triazole was the most potent with Ki value of 0.3 µM [66].

It is speculated that EHNA is the best compound to distinguish ADA isoenzymes. In many experimental studies, this molecule inhibited only ADA1 activity, while ADA2 remained resistant to the inhibition by EHNA as well as its derivatives [46]. Although, the similarities between the structure of ADA1 and ADA2 active centers have been shown, it has been proposed that the absence of hydrophobic site in ADA2 could prevent EHNA binding [12].

It has been shown however that EHNA is not a specific ADA1 inhibitor. It also blocks the activity of cyclic nucleotide phosphodiesterase 2 (PDE2), which belongs to the family of phosphohydrolases that selectively catalyze the hydrolysis of 3-cyclic phosphate bonds of adenosine and/or guanosine 3,5-cyclic monophosphates [90]. PDE2 possesses a low affinity catalytic domain and an allosteric domain specific for cGMP. The low affinity catalytic site can hydrolyze both cAMP and cGMP with a lower apparent Km for cGMP over cAMP. However, when cGMP binds to the allosteric site, the catalytic site undergoes a conformational change showing high affinity for cAMP. It has been demonstrated that EHNA inhibited PDE2 with an apparent Ki value of 1 µM and it has negligible effects on Ca^2+^/calmodulin PDE (PDE1), cGMP-inhibited PDE (PDE3), and low *K*_m_ cAMP-specific PDE (PDE4). The core structure of EHNA resembles cAMP but differentiates in the fact that EHNA has a bulky hydrophobic carbon side chain replacing the phospho-ribose moiety in cAMP [91]. In experimental studies on primary cultures of rat cortical neurons, the inhibition of PDE2A by EHNA potentiated *N*-methyl-d-aspartate (NMDA) receptor-activated increase in cGMP, but had no effect on cAMP concentrations [92]. In cardiovascular system, both cAMP and cGMP have an important role in the regulation of inotropic mechanisms in the human myocardium [93]. Therefore, the dual inhibition of PDE2 and ADA by EHNA could lead to accumulation of cGMP and adenosine, which may act in synergy to mediate diverse pharmacological responses including anti-arrhythmic effects. 

### 3.3. Non-Nucleoside Inhibitors

Despite the effectiveness of the compounds described above in the inhibition of ADA activity, they poor pharmacokinetics is still an obstacle to the routine use. However, recent progress has been achieved through the development of new non-nucleoside ADA inhibitors that include a series of imidazole-4-carboxamides [94]. Concentrating on molecular interactions between 1-deazaadenosine and murine ADA, Terasaka et al. designed compounds that retain the main sites of interaction with the enzyme [49]. This led to the synthesis of 1-(1-hydroxy-4-phenylbutan-2-yl)-1*H*-imidazole-4-carboxamide that exhibited good pharmacokinetic properties (oral bioavailability) and a Ki value of 5.9 µM [67]. Next, the authors sought more potent, orally active, compounds to inhibit ADA with a minimal cytotoxicity. Especially, 1-((1*R*,2*S*)-2-hydroxy-1-(2-(1-naphthyl)ethyl)propyl)-1*H*-imidazole-4-carboxamide (FR234938) with Ki = 3.6 nM, proved to be potent, competitive and not tight-binding ADA inhibitor. FR234938 showed an effective anti-inflammatory efficacy [68,94,95]. In the presence of sub-effective adenosine doses, it augmented IL-6 induced IgM production by human lymphoblastoid cells in vitro in a concentration-dependent manner. It has been shown that this effect was dependent on A2a adenosine receptors, since it was abolished by ZD4398, a selective A2a antagonist. The modulation of extracellular adenosine levels suggests that FR234938 effectively inhibited cell-surface eADA activity. FR234938 also significantly reduced pro-inflammatory cytokine production in mouse LPS model as well as decreased IL-10 and TNF-α concentration in mice after subcutaneous administration [68]. Therefore, it has been reasonably considered that imidazole-4-carboxamides are promising drug candidates for the treatment of pathological conditions with ADA overactivity, including discussed below cardiovascular diseases.

### 3.4. Flavonoids and Sapogenins/Plant Extracts

Many components of dietary crude plant extracts, such as onion and garlic extracts may exert biological activities through the inhibition of ADA. Among them, flavonoids and sapogenins exhibit attractive biological properties [74]. Flavonoids are the most common group of phenolic compounds in the human diet that are synthetized by vegetables, fruits and plants. These plant phenolic derivatives have many pharmacological effects, including the moderate inhibition of ADA activity. These compounds display competitive ADA inhibition and through the subsequent accumulation of endogenous adenosine would exert some beneficial effects. Studies of the inhibition structure-activity of these compounds for ADA have been undertaken and disclosed that hydroxyl group in 3 position of chromone moiety is needed for the inhibitory activity. Also hydroxyl groups in the side phenyl ring seem to be partly important. The inhibition of ADA by flavonoids was found to be both substrate and inhibitor concentration-dependent. Kaempferol and quercetin exhibited the IC_50_s of this inhibition about 30 µM [69]. Hibifolin isolated from *Helicteres isora* demonstrated the inhibitory constant equal to 50 µM [70]. The Ki values of naringrin were determined for various substrates for ADA, including adenosine, 2′deoxyadenosine and cordycepin (3′deoxyadenosine) and was about 200 µM [71]. Curcumin (diferuloylmethane), a flavonoid from the rhizome of *Curcuma longa* also inhibited ADA with an IC_50_ value of 13.6 µM [72]. Genistein from tofu wastewater and cyanidin-3-rutinoside from litchi peel had IC_50_ values for ADA inhibition of 1.5 mM and 0.95 mM, respectively. [73]. The effects of several sapogenins and saponins of the steroid and triterpenoid classes, as well as of a few common phytosterols, have been tested for their inhibitory actions towards adenosine deaminase by Koch et al. The authors have found that the *acidic* sapogenins potently inhibited the enzyme with Ki value estimated as about 1 µM. In contrast, *neutral* sapo(ge)nins and phytosterols did not affect ADA activity [74,96]. Recently, Zhang et al. isolated endophytes from six plants, which are widely used in Chinese herbal medicine for the screening of ADA inhibitors [75]. They identified a strong uncompetitive inhibitor of ADA activity, 3-(4-nitrophenyl)-5-phenylisoxazole, which demonstrated an IC_50_ value equal to 0.380 mM.

### 3.5. Clinically Used Drugs Not Targeting ADA

A number of frequently used drugs have a significant inhibitory effect on ADA activity in their therapeutic doses. Among them acetaminophen and non-steroid anti-inflammatory drugs, such as diclofenac and aspirin exhibited a Ki value of 214 µM, 30 µM and 43 µM, respectively [76,77]. Sedative, anxiolytic, analgesic, and relaxant drugs, including lidoflazine, phenylbutazone, chlordiazepoxide and tradozon showed a Ki value for ADA of 30, 54, 83 and 60 µM, respectively [78,79,97]. Kowalczyk et al. studied the influence of selected cardiovascular drugs on the activity of ADA in rabbits [98]. The authors showed that at therapeutic doses simvastatin inhibited tADA by 50%, metoprolol by 29%, and isosorbide mononitrate by 19%. Interestingly all analyzed drugs altered both ADA1 and ADA2 activities. Therefore, already used cardiovascular drugs could extend the half-life of endogenous adenosine by ADA inhibition, and this may partially explain some pleiotropic effects of these drugs.

### 3.6. The Challenges in Ecto-Adenosine Deaminase (eADA) Inhibition

The vast majority of identified ADA inhibitors show cell-permeable properties, causing inhibition of its intra- and extracellular activities. The consequence of the inhibition of intracellular ADA may be several adverse effects including cytotoxicity and immunosuppression (due to intracellular 2′-deoxyadenosine accumulation and suppression of DNA synthesis in actively dividing cells, such as lymphocytes), so it is particularly important to search for new inhibitors acting specifically on the eADA or approaches that block membrane transport of known ADA inhibitors. One of the possibilities to limit adenosine deaminase inhibition to the extracellular compartment is a combination of nucleoside-derived ADA inhibitors, such as 2′-deoxycoformycin or chloro-2′-deoxyadenosine with ENT1 nucleoside transport inhibitors (e.g., NBTI, dipyridamole, or ticagrelor) as both of these nucleoside derivatives are transported via ENT1. [99] The use of nucleoside transport inhibitors has been accepted as an extracellular adenosine-elevating strategy. Thus, protective or ameliorating effects of adenosine uptake inhibitors in ischemic cardiac and cerebral injury, organ transplantation, seizures, thrombosis, insomnia, pain, and inflammatory diseases have been reported [100]. Moreover ticagrelor, except inhibiting ENT1 transporter, is a novel class of antiplatelet drugs that inhibits platelet P2Y_12_ receptors [101]. While dipyridamole has additional ADA inhibition properties itself [102]. Interestingly, some ADA inhibitors can also suppress the concentrative nucleoside transport system (CNT) [103]. All of this underlines that combined therapy of ADA inhibitors with nucleoside transport inhibitors may have synergistic benefits resulting from the primary drug effect (as ticagrelor affects platelets) and the effects on the retention of adenosine and nucleoside-derived ADA inhibitors in the extracellular compartment, which could be especially important for the treatment of cardiovascular diseases [104]. 

## 4. Inhibitors of ADA Binding to Anchoring Proteins

It has been demonstrated that HIV-1 envelope protein gp120 inhibited the interaction between ADA and CD26 on the surface of human cells [105]. This inhibition was observed in human CD4^+^ and CD4^−^ cells, thus appearing to be independent of HIV infection and hence the interaction of HIV-1 gp120 with CD4, suggesting a direct interaction between gp120 and ADA binding site of CD26. Moreover, it has been reported that anti-ADA antibodies inhibited ADA binding to CD26, which suppressed co-stimulatory function of ADA protein and as a consequence impaired T-cell proliferation [106].

## 5. Cardiovascular Pathologies Associated with Increased ADA Activity as Potential Targets for ADA Inhibition

### 5.1. Atherosclerosis

Extracellular purines play a significant role in cardiovascular system homeostasis. Adenosine that is abundantly produced on the surface of endothelial and vascular smooth muscle cells by ecto-nucleotidases, dampens via P1 receptors endothelial activation, immune cell adhesion, smooth muscle cell proliferation and foam cell formation that all together initiate and drive atherosclerotic process in the vessel wall [4,107,108,109,110]. Under stress conditions related to atherosclerosis, such as hypoxia, depressed cellular energy state lead to acute increase and release of adenosine from the cell [111]. However, except intracellular adenosine metabolism and release, compensatory effects of adenosine in cardiovascular system are also regulated by other mechanisms, including cell-surface metabolism. In this context, vascular ecto-adenosine deaminase represents a critical checkpoint in the regulation of extracellular adenosine level in the vessel wall and, consequently, in the control of receptor stimulation, thus playing a pivotal role in the modulation of purinergic responses that lead to atherosclerosis development.

Overactivity of serum ADA has been described in patients with increased cardiometabolic risk and atherosclerosis. The correlation analysis of cardiometabolic syndrome risk factors against ADA also showed a strong association [112]. This is in line with recent results of Simard et al., where advancing age and the presence of obstructive coronary artery disease were associated with lower plasma adenosine levels [113]. The increase in serum ADA activity was also more pronounced in males than in females, reflecting a greater susceptibility of men to the development of atherosclerosis. In our study, we revealed that ADA activity was also augmented in atherosclerotic vessel wall [114]. Moreover, using a mouse model of atherosclerosis, we established that the increase in vascular eADA activity was much higher comparing to ADA activity in serum [115]. In the same study, we have shown that vascular eADA activity preceded and correlated with the development of atherosclerosis. Similarly, we revealed that in humans, vascular eADA activity positively correlated with endothelial activation markers, plasma triglycerides, LDL cholesterol and vessel lipid content [4]. We noticed that at the initial stages of atherosclerosis, vascular eADA originated from activated endothelial cells, while at later stages, it was derived from immune cells that infiltrated the vessel wall [115]. Therefore, vascular eADA could represent an interesting parameter that reflects the severity of endothelial activation and vascular inflammation. Recently, we observed that the activation of JAK/STAT pathway stimulated ADA expression in endothelial cells. Whereas transcytosis pathways, in particular cholesterol-dependent exocytosis via lipid rafts, have been responsible for ADA externalization on the cell surface [4].

It has been demonstrated that increased activity of eADA induced hyperpermeability of endothelial cells and can stimulate interactions between endothelial and immune cells through enzymatic and extra-enzymatic properties [106,116,117]. In the first case, lymphocytes adhesion to endothelium was controlled via the modulation of vascular adhesion molecule expression by decreased adenosine receptor signaling [118]. While in the second case, this interaction did not result from the enzymatic activity of ADA or CD26, but rather from the interaction of the complex, expressed on T cell surface, and an ADA1-anchoring protein located in endothelial cell [119]. This co-stimulatory signal led to a marked increase in the production of T helper-1 (Th1) cells and Th1 proinflammatory cytokines, such as interleukin-6 (IL-6), interferon (IFN)-γ and tumor necrosis factor-α (TNF-α) [120]. It has been showed that these cytokines prompted plaque progression and that Th1 cells exceeded the number of athero-protective Th2 cells in atheroma regions. Other studies have shown an involvement of adenosine in lymphocyte proliferation and Th1/Th2 cytokine production and differentiation, thus confirming a critical role played by the activity of eADA in lymphocyte-dependent atherosclerosis progression through the modulation of endogenous nucleoside levels [121].

Another substantial role of ADA in the modulation of immune response that leads to atherosclerosis is differentiation of monocytes to macrophages with subsequent macrophage proliferation dependent on Th cells. It has been suggested that this process is also mediated by extra-enzymatic activity and requires ADA2 as a member of ADA growth factor family (ADGF). Antigen presenting cells (APC), such as monocytes exclusively secreted ADA2 protein that bound to proteoglycans expressed on their surface. In turn, T cells bound ADA2 via more specific receptors leading to the creation of a “molecular bridge” between APC and T cell that activated Th cell-induced differentiation of monocytes to macrophages [122]. A similar effect was observed when monocytes were grown in the presence of endothelial cells suggesting co-stimulation between APC and endothelial cell via monocyte-released ADA2. [123] ADA2 seems to be also involved in the balance between pro-inflammatory (M1) and anti-inflammatory (M2) macrophages, since it plays a key role in M2 macrophage differentiation. Its absence has been in fact associated with a defect in the formation of M2 macrophages, which leads to a prevalence of pro-inflammatory M1 cells [124]. However, the role of macrophage phenotype in atherosclerosis is not fully elucidated. It has been demonstrated that both M1 and M2 macrophages are present in atherosclerotic plaques. De Gaetano et al. revealed that M2 macrophages were found in more stable locations within the lesions, while the relative percentage of M1 macrophages was significantly higher in symptomatic plaques. [125] These observations suggests that plaque instability might be caused by an imbalance between M1 and M2 macrophages and their polarization towards an M1 phenotype. Indeed, M1 macrophages have significant roles in plaque progression, monocyte recruitment into plaques, and vulnerable plaque development [126]. While M2 macrophages may have predominantly anti-atherogenic functions, some of their properties may promote plaque progression, especially in terms of sensitivity to oxLDL uptake, which may promote enlargement of the core. Tits et al. showed that M2 macrophages were more susceptible to foam cell formation than M1 subtype and exposure to oxidized LDL rendered M2 macrophages pro-inflammatory, driving atherosclerosis process [127]. Therefore, extra-enzymatic role of ADA2 cannot be unambiguously assigned in atherosclerosis progression by controlling of macrophage polarization. 

Nonetheless, the activity of eADA could be considered as an important regulator of macrophage functions in atherosclerosis. It may stimulate macrophage transformation to foam cells and upregulates reverse cholesterol transport proteins: cholesterol 27-hydroxylase and ATP binding cassette transporter A1 (ABCA1) via extracellular adenosine deprivation. Reiss et al. determined that adenosine via A2a receptors affected expression of proteins involved in cholesterol flux in monocytes/macrophages and, consequently, provided a defense against lipid overload-induced macrophage foam cell formation [128].

Together, enzymatic and extra-enzymatic properties of ADA, especially its ecto-form, could be considered as pro-atherosclerotic and their inhibition deserves special attention in cardiovascular preventing therapy. In mouse model of severe atherosclerosis, we determined anti-atherosclerotic effect of low dose dCF that inhibited vascular eADA activity by more than 80%. This effect was related to endothelial protection and anti-inflammatory properties of adenosine [115]. It has been shown that adenosine deaminase inhibition decreased the expression of adhesion molecules and pro-inflammatory cytokine release by endothelial cells [118]. Previously, we revealed that dCF protected against LPS-induced endothelial activation via the increase in extracellular adenosine level and further adenosine receptor-dependent effects [4]. Since most of the protective effects of ADA inhibition in vasculature have been achieved by the suppression of its cell-surface activity, specific inhibitors for eADA, free of side effects caused by the influence on its intracellular activity are urgently needed.

### 5.2. Thrombosis

Platelet hyper-aggregability is one of the major risk factors for cardiovascular disease. The accumulation of platelets at vascular injury sites is the primary event in arterial thrombosis and its activation is a critical component of atherothrombosis [129]. It has been described that patients with unstable atherosclerotic lesions demonstrated a significantly higher platelet activation than patients with stable angina, indicating an intense thrombogenic potential [130]. Extracellular adenosine by both A2 receptors, A2_A_ and A2_B_ that are coupled to Gs, leads to the stimulation of adenylyl cyclase and consequent elevation of cAMP, the best-known inhibitor and turn off signaling in platelet activation [131]. Therefore, extracellular adenosine metabolism that regulates adenosine availability for its receptors plays a pivotal role in the anti-aggregatory properties of this nucleoside. Indeed, ecto-enzymes engaged in adenosine metabolism have been recognized on the surface of platelets, including ATP-degrading ecto-nucleoside triphosphate diphosphohydrolase (eNTPDase) and ecto-nucleotide pyrophosphatase/phosphodiesterase (eNPP), adenosine-producing ecto-5′-nucleotidase (e5′NT) and adenosine-degrading eADA [132]. Suoza Vdo et al. revealed increased activities of eNPP and e5′NT and decreased eADA activity in platelets of patients with Chagas disease contributed to decreased platelet aggregation, suggesting that the purinergic system is significantly involved in the thromboregulation [132], whereas, Leal et al. demonstrated that serum ADA activity positively correlated with increased platelet aggregation in pregnant women with an increased risk of cardiovascular disease [133]. Therefore, the increase in ADA activity can contribute to adenosine depletion followed by the activation of a pro-aggregatory phenotype. Stafford et al. have shown that ATP-stimulated platelet aggregation, which was induced via ATP to ADP conversion by ecto-ATPases and further activation of P2 receptors on platelets was potentiated by removal of adenosine by eADA [134]. Furthermore, ADA reversed dipyridamole-inhibited platelet aggregation that decreased adenosine uptake into erythrocytes [134]. This indicates that ADA activity poses an essential role in the downregulation of anti-aggregatory adenosine and ADA inhibitors could serve as anti-platelet drugs. However, Fuentes et al. revealed that inosine was also capable to inhibit platelet adhesion and aggregation under flow and thrombus growth in vivo [135]. Although inosine, in contrast to adenosine, did not interact with platelet receptors, both nucleosides inhibited P-selectin expression on human platelets induced by ADP/collagen. The authors suggested that adenosine and inosine could inhibit platelet-leukocyte conjugate formation [44]. However, eADA may affect interactions between platelets and leukocytes also via its extra-enzymatic properties functioning as an adhesion molecule. The simultaneous presence of adenosine receptors (AR) on the platelets together with eADA activity suggests that AR are responsible for ADA anchoring on the platelet surface. In this sense, all cell types with abundant expression of CD26 protein are prone to binding to platelets through CD26-ADA-AR complex [6]. This particularly applies to the interactions of platelets with endothelial and immune cells that contribute to endothelial injury, thrombosis, and inflammation [136]. Taken together, the inhibition of ADA activity and further activation of AR on platelets as well as the suppression of ADA binding to the platelets have therapeutic potential in coagulation disorders, but the effects of pharmacological intervention with ADA inhibitors in these pathological conditions require further studies.

### 5.3. Acute Myocardial Infarction and Myocardial Ischemia-Reperfusion Injury

It has been demonstrated that patients with acute myocardial infarction (AMI) revealed higher activity of serum ADA than age-matched controls [137]. In the study provided by Patil et al., serum ADA activity has been recognized as a marker of an AMI-related inflammation [138]. Indeed, the main mechanism of reperfusion injury during AMI is an inflammatory response that includes the accumulation of neutrophils. Adenosine that inhibit the neutrophil adhesion and exudation, can reduce the reperfusion injury [139]. More precisely, adenosine is a strong inhibitor of neutrophil respiratory burst, preventing neutrophil adherence and cytotoxicity to the endothelial cells [140]. Also macrophages, particularly M1 subtype, are involved in myocardial ischemia-reperfusion (IR) injury. It has been indicated that adenosine protected the heart against IR injury by the modulation of macrophages phenotype into M2 subset through a PI3K/Akt pathway [141]. Other studies confirmed the role of M2 macrophages in the reduction of reperfusion injury, neoangiogenesis stimulation and scar repair after myocardial ischemia via anti-inflammatory and pro-angiogenic properties mediated by IL-10, vascular endothelial growth factor, and TGF-β1 [142]. Since shifting the balance from M1 to M2 macrophages improves myocardial repair, extra-enzymatic function of ADA2 in M2 macrophage differentiation seems to have a protective significance. However, overactivity of ADA leading to insufficient adenosine level could counteract its protective effects on the reperfusion injury brought by neutrophils and macrophages. 

The activity of ADA is also engaged in the ischemic reperfusion injury caused by superoxide radicals via the regulation of both, adenosine and inosine concentration. Adenosine is known to reduce the injury caused by superoxide radicals [143]. While, inosine is further metabolized to hypoxanthine that is involved in the superoxide formation [144]. Consequently, increased activity of ADA could stimulate reperfusion injury induced by superoxide radicals via decreased level of protective adenosine and increased level of superoxide radicals-producing inosine. 

Torrellas et al. provided a study where patients with an AMI had an elevated activity of serum ADA at the time of hospital admission, which decreased after 24 and 72 h of treatment onset. The authors suggested that an elevated serum ADA activity in patients diagnosed with AMI could be a consequence of myocardial and/or pulmonary hypoxia [145]. Since oxygen deprivation that occurs during hypoxia is a strong inductor of adenosine release that plays a key role as a cardioprotective mediator of pre- and post-conditioning, the increased ADA activity could endure adenosine-dependent cardioprotection.

The significant role of ADA has been also demonstrated in the coronary vasodilatory response to systemic hypoxia. It has been shown that ADA infusion into the left anterior descending artery (LAD) significantly decreased coronary blood flow induced by severe hypoxia. Oxygen consumption in the LAD perfusion field was unchanged by hypoxia before ADA but fell significantly during hypoxia after ADA. ADA also attenuated significantly the coronary vasodilatory response to exogenous adenosine and to 20-s ischemia [146].

It has been revealed that ADA is also involved in the arrhythmia. In isolated perfused rat hearts with occlusion of the left coronary artery that were treated with exogenous ADA, the release of adenosine and its degradation products inosine, hypoxanthine, xanthine and uric acid has been measured. It was demonstrated that reperfusion-induced ventricular fibrillation was associated with an increase in the release of adenine nucleotide catabolites, compared with non-fibrillating hearts. In ADA treated hearts the incidence of reperfusion induced fibrillation increased, thereby contributes to the enhanced release of adenine nucleotide catabolites [147].

ADA inhibition has also been proposed as part of adenine nucleotide pool replenishment strategy in post ischemic cardiomyocytes [148]. Protection from reperfusion injury after cardiac transplantation by inhibition of adenosine metabolism and nucleotide precursor supply [149]. Such approach used adenine, ribose and adenosine kinase inhibitor together with inhibition of ADA. This allowed to increase production of adenosine without deterioration of adenine nucleotide pool in the cells. Use of ADA inhibition together with adenine, ribose and adenosine kinase inhibition reduced post ischemic injury in transplanted hearts.

Summarizing, ADA could be a promising therapeutic target in myocardial ischemia-reperfusion injury. It should be noted however that inhibiting all of its properties may have not a satisfactory effect. Certainly, inhibition of ADA activity may be beneficial in retaining protective functions of adenosine but extra-enzymatic properties exerted especially by monocyte-released ADA2 should be unaffected. Experimental evidence for beneficial effects of ADA inhibition therapy in myocardial ischemia has been provided by McClanahan et al. [150]. In a dog model of myocardial ischemia, all animals underwent 15 min of coronary occlusion followed by 3 h of reperfusion preceded by an intravenous bolus of either dCF or saline. The dCF group demonstrated better contractile function at all time points during reperfusion by augmenting endogenous extracellular adenosine levels. These results indicate that the inhibition of ADA activity can ameliorate the severity of myocardial post-ischemic contractile dysfunction.

### 5.4. Hypertension

The increase in ADA activity has been also described in hypertension. Rao et al. demonstrated higher serum ADA activity in gestational hypertensive women than in normotensive pregnant women [151]. In other study, Sajjan et al. showed a significant positive correlation between enhanced serum ADA levels and increase in systolic (SBP) and diastolic blood pressure (DBP) in subjects with metabolic syndrome [112]. Also our results revealed a strong positive correlation of tissue eADA activity estimated in calcified aortic valves with SBP and DBP in patients with calcified aortic valve disease [24]. There is no data elucidating the origin of elevated ADA activity in hypertension. However, shear stress that accompanied increased blood pressure predisposes to endothelial injury and further infiltration of immune cells into a subendothelial layer [152]. As we demonstrated previously, both activated endothelial and immune cells represent an abundant source of ADA that may explain its increased activity in hypertensive patients [153]. It has been investigated that systemic adenosine via an A1 receptor calcium-mediated pathway inhibited renin release from juxtaglomerular cells [154]. Thus, as it has been shown by Kaun et al., decreased extracellular adenosine concentration caused by augmented eADA activity led to a positive regulation of renin secretion and elevated plasma renin activity following by increased blood pressure and hypertension [155]. Tofovic et al. determined the beneficial effects of ADA inhibition in hypertension by the modulation of endogenous adenosine levels in hypertensive rats [156]. ADA inhibition by EHNA produced a marked fall in arterial blood pressure in old spontaneously hypertensive rats, whereas no effect on blood pressure were observed in age-matched normotensive Wistar Kyoto rats. Adenosine receptor antagonist, DPSPX blocked the antihypertensive effects of EHNA, proving that the effects of EHNA on blood pressure were mediated by peripheral adenosine receptors [156].

### 5.5. Type 2 Diabetes Mellitus

Type 2 diabetes mellitus (T2DM) is associated with increased risk for cardiovascular disease, which is often fatal among diabetics. Metabolic disturbance and immunological imbalance are two essential causes of T2DM. The most important metabolic factors include insulin resistance and insulin deficiency, while immunological disturbances in T2DM have been associated with inappropriate T-lymphocyte function and cell-mediated immunity [157]. ADA activity has been considered as a marker of cell-mediated immune response and its increase has been reported in serum of patients with T2DM [158]. Several reports have shown heightened activities of both ADA iso-enzymes during T2DM with values increasing in uncontrolled compared to controlled T2DM [159,160]. Takhshid et al. revealed that a polymorphism in the *ADA* gene that encodes ADA1 was associated with impaired glucose metabolism, poor glycemic control, and obesity in patients with gestational diabetes mellitus. Individuals with GG genotype exhibited increased enzymatic ADA activity that affected plasma glucose concentration and weight gain [161]. ADA was described as an important modulator of glucose metabolism in different tissues and its increased activity may be a direct cause of insulin resistance. Higher activity of ADA in insulin-sensitive tissues may reduce glucose uptake into cells through decreased adenosine level [162]. Adenosine via its receptors acts directly on tissues to stimulate insulin activity by increased glucose transport but also through lipid synthesis, pyruvate dehydrogenase (PDH) activity, leucine oxidation and cyclic nucleotide phosphodiesterase activity [163]. Hence, if ADA activity is inhibited, insulin sensitivity may be improved, and processes associated with the pathophysiology of insulin resistance such as cellular proliferation, inflammation, and T-cell activity can also be affected. Indeed, dCF treatment exhibited protective effects against insulin-dependent diabetes mellitus in the BB Wistar rats [164].

Next to the key role of ADA in T-cell activity and insulin resistance, it is bound on the cell surface by CD26 protein (DPP-4) that is an important modulator of insulin secretion. [165] DPP-4 (dipeptidyl peptidase-4) poses an enzymatic activity of ectopeptidase and rapidly degrades various peptides, including glucagon-like peptide-1, an incretin that promotes insulin secretion by pancreatic beta cells, inhibits glucagon secretion in alpha cells, decreases the gastric discharge rate, and mediates appetite suppression [166]. CD26 is abundantly expressed on mammalian endothelial, epithelial and immune cells and represents increased levels in T2DM alongside with ADA. Since, DPP-4 inhibitors are recognized as anti-diabetic compounds, their impact on ADA activity has been evaluated. Lee et al. showed no additional specific effects of CD26 inhibition on ADA activity, except for a glycemic control related to HbA1c decrease [167]. There are no reports on the effects of CD26 inhibitors on CD26-ADA binding, but due to a distance between catalytic site and ADA-binding site in a large extracellular domain of CD26 protein, it seems that CD26 inhibitors should not interfere with this binding [165]. Therefore, costimulatory signaling between CD26 and ADA would be preserved facilitating further T cell activation and T cell-dependent complications of diabetes [158]. Summarizing, the inhibition of eADA activity, simultaneously with the suppression of CD26-ADA binding and CD26 activity can be potentially effective against T2DM progression.

## 6. Conclusions

Adenosine deaminase is a moonlighting protein that exhibits intracellular and extracellular enzymatic activity, which modulates adenosine and 2′-deoxyadenosine concentration inside and outside the cell. Moreover, it has extra-enzymatic, co-stimulatory properties. Therefore, adenosine deaminase is an essential regulator of multiple cellular processes, including DNA synthesis, adenosine receptor-mediated pathways and cell-to-cell interactions. Overactivities of total-, soluble- and ecto- adenosine deaminase have been described in many cardiovascular pathologies (Table 2) including atherosclerosis, thrombosis, hypertension, myocardial ischemia-reperfusion injury, and diabetes, stimulating their further development. Therefore, the inhibition of adenosine deaminase expression and activity (particularly its cell-surface and soluble forms) has been proposed as a promising cardioprotective therapy. Although the most popular adenosine deaminase inhibitors, such as 2′deoxycoformycin and EHNA exhibited beneficial effects for the development and progression of cardiovascular diseases, they have problems with poor pharmacokinetics and several toxicities related to their permeability through the cell membrane. So far, 2′deoxycoformycin is the only adenosine deaminase inhibitor in clinical use that currently is limited to the treatment of adult patients with hairy cell leukemia. Unfortunately, its acid-lability results in the lack of oral bioavailability and imposes its intravenous administration. Therefore, the development of novel cell non-permeable adenosine deaminase inhibitors that would have adequate pharmacokinetic properties is urgently needed.

## Figures and Tables

**Table 1:** The main classes of adenosine deaminase inhibitors with inhibitory constant (Ki) value or the half maximal inhibitory concentration (IC_50_).

Adenosine Deaminase Inhibitor	Ki Value (or IC_50_)	Ref.
Transition-state inhibitors		
Coformycin	10 pM	[50]
2′-Deoxycoformycin	2.5 pM	[51]
4*R*-(1-Hydroxyethyl)-5-methyl-1-β-D-ribofuranosylimidazole	61 µM	[52]
2′-Chloro-2′deoxyadenosine (cladribine)	(0.2 µM)	[53,54]
2′-Chloro-2′deoxycoformycin	0.53 nM	[55]
Adecypenol	47 nM	[56]
**Ground-state inhibitors**		
1-Deazaadenosine	0.66 µM	[57]
2′-Deoxy-1-deazaadenozine	0.19 µM	[58]
N^6^-Hydroxy-2′deoxy-1-deazaadenozine	0.25 µM	[59]
N^6^-Methyl-2′deoxy-1-deazaadenozine	1.2 µM	[59]
N^6^-Cyclopropyl-2′deoxy-1-deazaadenozine	5.9 µM	[59]
3′-Deoxy-1-deazaadenosine	2.6 µM	[60]
2′3′-Dideoxy-1-deazaadenosine	2.2 µM	[60]
Erythro-9-(2-hydroxy-3-nonyl) adenine (EHNA)	1.6 nM	[61]
9′-Chloro-EHNA	2.7 nM	[62]
9′-Phthalimido-EHNA	0.95 nM	[62]
Fluorescent derivatives of epsilon-EHNA	2.8 µM	[63]
1-DeazaEHNA	0.16 µM	[64]
3-DeazaEHNA	0.01 µM	[64]
Erythro-1-(2-hydroxy-3-nonyl)imidazole	0.90 µM	[65]
Erythro-9-(2-hydroxy-3-nonyl)imidazole-4-carboxamide	0.035 µM	[65]
Erythro-9-(2-hydroxy-3-nonyl)1,2,4-triazole	0.3 µM	[66]
**Non-nucleoside inhibitors**		
1-(1-Hydroxy-4-phenylbutan-2-yl)-1*H*-imidazole-4-carboxamide	5.9 µM	[67]
1-((1*R*,2*S*)-2-Hydroxy-1-(2-(1-naphthyl)ethyl)propyl)-1*H*-imidazole-4-carboxamide (FR234938)	3.6 nM	[68]
Flavonoids and sapogenins/plant extracts		
Kaempherol	(30 µM)	[69]
Quercetin	(30 µM)	[69]
Hibifolin	50 µM	[70]
Naringrin	200 µM	[71]
Curcumin	(13.6 µM)	[72]
Genistein	(1.5 mM)	[73]
Cyanidin-3-rutinoside	(0.95 mM)	[73]
*Acidic* sapogenins 3-(4-Nitrophenyl)-5-phenyl isoxazole	1 µM (0.380 mM)	[74] [75]
Drugs		
Acetaminophen	214 µM	[76]
Diclofenac	30 µM	[77]
Aspirin	43 µM	[77]
Lidoflazine	30 µM	[78]
Phenylbutazone	54 µM	[78]
Chlordiazepoxide	83 µM	[78]
Tradozon	60 µM	[79]

**Table 2:** Cardiovascular diseases with ADA overactivity and therapeutic effects of its inhibition in experimental models. AMI—acute myocardial injury; IRI—ischemia-reperfusion injury; T2DM—type 2 diabetes mellitus; tADA—total adenosine deaminase; ADA1—adenosine deaminase 1; ADA2—adenosine deaminase 2; dCF—2′deoxycoformycin; EHNA—erythro-9-(2-hydroxy-3-nonyl) adenine; n.d.—no data.

Cardiovascular Pathology	ADA Activity	ADA Inhibitor	Therapeutic Effect of ADA Inhibition
Atherosclerosis	↑ tADA (plasma) [112] ↑ ADA1 (vessel wall) [115]	dCF [115]	+
Thrombosis	↑ tADA (plasma) [134]	n.d.	n.d.
AMI/IRI	↑ tADA (plasma) [138,139]	dCF [151]	+
Hypertension	↑ tADA (plasma) [112]	EHNA [157]	+
T2DM	↑ tADA (plasma) [159] ↑ ADA1 (plasma) [160,161] ↑ ADA2 (plasma) [160,161]	dCF [165]	+
